# Increased local cytostatic drug exposure by isolated hepatic perfusion: a phase I clinical and pharmacologic evaluation of treatment with high dose melphalan in patients with colorectal cancer confined to the liver

**DOI:** 10.1054/bjoc.2000.1175

**Published:** 2000-04-03

**Authors:** A L Vahrmeijer, J H van Dierendonck, H J Keizer, J H Beijnen, R A E M Tollenaar, M E J Pijl, A Marinelli, P J K Kuppen, J H van Bockel, G J Mulder, C J H van de Velde

**Affiliations:** Departments of Surgery, Clinical Oncology, Radiology, Leiden University Medical Center, PO Box 9600, Leiden, RC 2300, The Netherlands; Division of Toxicology, Leiden/Amsterdam Center for Drug Research, PO Box 9503, Leiden, RA 2300, The Netherlands; Faculty of Pharmacy, University Utrecht, Netherlands Cancer Institute/Slotervaart Hospital, Louwesweg 6, Amsterdam, EC 1066, The Netherlands

**Keywords:** isolated hepatic perfusion, melphalan, colorectal cancer, liver metastases

## Abstract

A phase I dose-escalation study was performed to determine whether isolated hepatic perfusion (IHP) with melphalan (L-PAM) allows exposure of the liver to much higher drug concentrations than clinically achievable after systemic administration and leads to higher tumour concentrations of L-PAM. Twenty-four patients with colorectal cancer confined to the liver were treated with L-PAM dosages escalating from 0.5 to 4.0 mg kg^−1^. During all IHP procedures, leakage of perfusate was monitored. Duration of IHP was aimed at 60 min, but was shortened in eight cases as a result of leakage from the isolated circuit. From these, three patients developed WHO grade 3–4 leukopenia and two patients died due to sepsis. A reversible elevation of liver enzymes and bilirubin was seen in the majority of patients. Only one patient was treated with 4.0 mg kg^−1^L-PAM, who died 8 days after IHP as a result of multiple-organ failure. A statistically significant correlation was found between the dose of L-PAM, peak L-PAM concentrations in perfusate (R = 0.86, *P*≤ 0.001), perfusate area under the concentration-time curve (AUC; R = 0.82, *P*< 0.001), tumour tissue concentrations of L-PAM (R = 0.83, *P* = 0.011) and patient survival (R = 0.52, *P* = 0.02). The peak L-PAM concentration and AUC of L-PAM in perfusate at dose level 3.0 mg kg^−1^(*n* = 5) were respectively 35- and 13-fold higher than in the systemic circulation, and respectively 30- and 5-fold higher than reported for high dose oral L-PAM (80–157 mg m^−2^) and autologous bone marrow transplantation. Median survival after IHP (*n* = 21) was 19 months and the overall response rate was 29% (17 assessable patients; one complete and four partial remissions). Thus, the maximally tolerated dose of L-PAM delivered via IHP is approximately 3.0 mg kg^−1^, leading to high L-PAM concentrations at the target side. Because of the complexity of this treatment modality, IHP has at present no place in routine clinical practice. © 2000 Cancer Research Campaign


British Journal of Cancer (2000) **82**(9), 1539–1546
© 2000 Cancer Research Campaign
DOI: 10.1054/ bjoc.2000.1175, available online at http://www.idealibrary.com on IDEAL®

### Increased local cytostatic drug exposure by isolated hepatic perfusion: a phase I clinical and pharmacologic evaluation of treatment with high dose melphalan in patients with colorectal cancer confined to the liver

**AL Vahrmeijer[1,4], JH van Dierendonck[1], HJ Keizer[2], JH Beijnen[5], RAEM Tollenaar[1], MEJ Pijl[3], A Marinelli[1], PJK Kuppen[1], JH van Bockel[1], GJ Mulder[4] and CJH van de Velde[1]**

Departments of [1]Surgery, [2]Clinical Oncology and [3]Radiology, Leiden University Medical Center, PO Box 9600, 2300 RC, Leiden, The Netherlands; [4]Division of Toxicology, Leiden/Amsterdam Center for Drug Research, PO Box 9503, 2300 RA, Leiden, The Netherlands; [5]Faculty of Pharmacy, University Utrecht, Netherlands Cancer Institute/Slotervaart Hospital, Louwesweg 6, 1066 EC, Amsterdam, The Netherlands

**Summary** A phase I dose-escalation study was performed to determine whether isolated hepatic perfusion (IHP) with melphalan (L-PAM) allows exposure of the liver to much higher drug concentrations than clinically achievable after systemic administration and leads to higher tumour concentrations of L-PAM. Twenty-four patients with colorectal cancer confined to the liver were treated with L-PAM dosages escalating from 0.5 to 4.0 mg $kg^{-1}$. During all IHP procedures, leakage of perfusate was monitored. Duration of IHP was aimed at 60 min, but was shortened in eight cases as a result of leakage from the isolated circuit. From these, three patients developed WHO grade 3–4 leukopenia and two patients died due to sepsis. A reversible elevation of liver enzymes and bilirubin was seen in the majority of patients. Only one patient was treated with 4.0 mg $kg^{-1}$ L-PAM, who died 8 days after IHP as a result of multiple-organ failure. A statistically significant correlation was found between the dose of L-PAM, peak L-PAM concentrations in perfusate (R = 0.86, $P \leq 0.001$), perfusate area under the concentration-time curve (AUC; R = 0.82, $P < 0.001$), tumour tissue concentrations of L-PAM (R = 0.83, $P = 0.011$) and patient survival (R = 0.52, $P = 0.02$). The peak L-PAM concentration and AUC of L-PAM in perfusate at dose level 3.0 mg $kg^{-1}$ ($n = 5$) were respectively 35- and 13-fold higher than in the systemic circulation, and respectively 30- and 5-fold higher than reported for high dose oral L-PAM (80–157 mg $m^{-2}$) and autologous bone marrow transplantation. Median survival after IHP ($n = 21$) was 19 months and the overall response rate was 29% (17 assessable patients; one complete and four partial remissions). Thus, the maximally tolerated dose of L-PAM delivered via IHP is approximately 3.0 mg $kg^{-1}$, leading to high L-PAM concentrations at the target side. Because of the complexity of this treatment modality, IHP has at present no place in routine clinical practice. © 2000 Cancer Research Campaign

**Keywords:** isolated hepatic perfusion; melphalan; colorectal cancer; liver metastases

Complete vascular isolation for in situ perfusion of organs or body parts provides interesting opportunities for cancer treatment. When properly performed, i.e. when leakage to the systemic circulation is avoided, delivery of anticancer drugs via an isolated circuit may have the obvious advantage that compounds and/or dosages can be used that would cause fatal complications if delivered systemically. In the treatment of irresectable hepatic metastases derived from colorectal cancer – a tumour type known to be quite resistant to systemic anticancer treatment – isolated hepatic perfusion (IHP) was already attempted clinically in the 1980s (Aigner et al, 1988). It was anticipated that by this approach a larger array of cytotoxic compounds could be tested, especially drugs with a steep dose–response relationship and with a higher toxicity to tumour cells than to surrounding liver tissue. IHP has been tested with drugs like melphalan (L-PAM) (Hafstrom et al, 1994), mitomycin C (MMC) (Marinelli et al, 1996), cisplatin (Hafstrom et al, 1994), 5-fluorouracil (Aigner et al, 1988) and tumour necrosis factor (TNF) (Borel Rinkes et al, 1997; Alexander et al, 1998). Recently, several clinical trials (Alexander et al, 1998; Oldhafer et al, 1998) have been started, exploring the efficacy of IHP with TNF and L-PAM under mild hyperthermic conditions to treat unresectable cancers confined to the liver (most of colorectal origin).

To date the number of drugs considered for IHP studies is still very limited, as these agents need to be effective after a single exposure (lasting no longer than the perfusion duration believed to be maximally obtainable without complications). Alkylating agents like MMC and L-PAM are effective against colorectal cancer after relatively short exposure times (Marinelli et al, 1991*a*, 1991*b*) and it is believed that above a certain threshold concentration, a relatively small increase in local drug concentration may result in a dramatic increase in tumour cell kill (Garcia et al, 1988). A high response rate (45%) has been reported for high dosed L-PAM in combination with autologous bone marrow transplantation as treatment for metastatic colon carcinoma (Leff et al, 1986). Whereas in the latter study no serious hepatotoxicity was observed, veno-occlusive disease (VOD) of the liver has been a recurrent problem after IHP with MMC (Marinelli et al, 1996; Oldhaffer et al, 1998). Moreover, comparing in a rat model for colorectal cancer hepatic metastases IHP with MMC, 5-fluorouracil or L-PAM, we found

*Received 28 July 1999*
*Revised 10 January 2000*
*Accepted 25 January 2000*

*Correspondence to:* CJH van de Velde that only with L-PAM complete remissions were obtained in all rats (Marinelli et al, 1991*a*, 1991*b*). These findings formed a sound rationale to start a phase I dose-finding study of IHP with L-PAM, which was performed during 1991–1994 in 24 patients. Because no relevant data had been published before on the pharmacokinetics of L-PAM in IHP setting, we sampled from all patients samples from the systemic circulation and isolated circuit, and, when possible, tumour and/or liver biopsies to determine L-PAM concentrations.

### PATIENTS AND METHODS

### Patient eligibility

Between May 1991 and March 1994, 24 patients with colorectal cancer confined to the liver were treated with a 60-min IHP using L-PAM dosages (Wellcome Pharmaceuticals B.V., Utrecht, The Netherlands) escalating from 0.5 to 4.0 mg $kg^{-1}$. This study was approved by the medical ethical committee of the Leiden University Medical Center, and informed consent was obtained from all patients. Selection criteria for IHP treatment were that no primary colorectal tumour was left and that metastases were confined to the liver and totally irresectable. Therefore, all patients were analysed using abdominal/chest computerized tomography (CT) and magnetic resonance imaging (MRI) scans. Eligibility criteria included a WHO performance status of 0 or 1, a leucocyte count $\geq 3.0 \times 10^9$ $l^{-1}$, a platelet count $\geq 100.0 \times 10^9$ $l^{-1}$, a serum creatinine level <135 μmol $l^{-1}$, a bilirubin level <17 μmol $l^{-1}$, an albumin level >40g $l^{-1}$ and with no coagulation disorders. Patients were excluded who had more than 75% hepatic replacement by tumour tissue or evidence of malignant ascites.

### IHP technique

The IHP technique with intra-caval double lumen shunt was used for the first 19 patients as previously described (Marinelli et al, 1996). Through a transversal abdominal incision the liver was first mobilized from the diaphragm and identifiable diaphragm veins were ligated. The pericardium was opened and the caval vein was dissected. After heparinization of the blood, the intrahepatic caval vein was cannulated with a specially designed double lumen catheter (Braun Melsungen, Germany). The inner lumen of this catheter allowed undisturbed blood flow to the right atrium of the heart, whereas the outer lumen served as a reservoir from which the hepatic venous outflow returned to a Cobe VPCML oxygenator (Cobe Cardiovascular, Inc, Arvada, CO, USA). The priming volume of the IHP system consisted of 1200 ml Gelofusine (Vifor Medical SA, Sempach, Switzerland) containing 2500 IE heparin. The mesenteric venous blood was drained to the inner lumen of the double lumen catheter by a temporary porto-caval shunt, which was established by cannulating the distal portal vein and connecting the catheter with the corresponding nozzle of the double lumen catheter. The two inflow limbs of the isolated circuit were both connected to two independent oxygenators (Custom-made liver perfusion tubing pack, Cobe Laboratories, Ltd, Gloucester, UK) and roller pumps (Cobe/Stöckert, model 10–30–00, Munich, Germany). One catheter entered into the portal vein and another through the gastroduodenal artery into the common hepatic artery. To isolate the hepatic circuit, tourniquets were secured around the caval vein above and below the incision, above the renal veins and within the pericardium. The celiac axis and the common bile duct were clamped. Throughout the perfusion period, the perfusate was kept at 37–38°C.

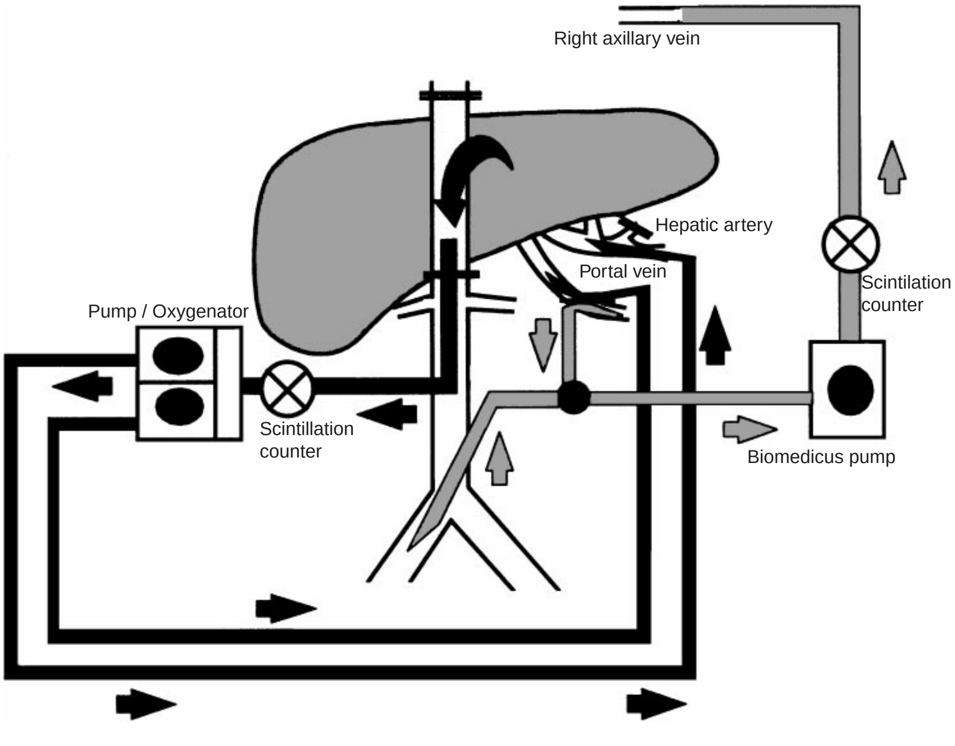

**Figure 1** Isolated hepatic perfusion circuit with extra-corporeal veno-venous bypass

 © 2000 Cancer Research Campaign

For the last five patients of this study, we used a different IHP technique (Vahrmeijer et al, 1996) with extracorporeal veno-venous bypass, supported by a Biomedicus centrifugal pump (Medtronic Bio-Medicus, Inc., Eden Prairie, MN, USA) (Figure 1). The right femoral vein was cannulated as well as the portal vein (proximal of the clamp) and connected to the right axillary vein (Gott, 7 mm cannula, Argyle, Sherwood Medial, St Louis, MO, USA). The system was primed with 700 ml saline (0.9%). The blood flow through the veno-venous bypass was approximately 21 $min^{-1}$. Systemic hypothermia throughout the perfusion period was prevented by the application of a heat-exchanger (Avecor Cardiovascular, Inc., Plymouth, MN, USA) which was placed between the tubing of the veno-venous bypass. To isolate the liver, the hepatic artery and portal vein were cannulated as described above. In contrast to the above-described technique, the caval vein was clamped above the right renal vein and below the diaphragm. The intrahepatic caval vein was cannulated (Polystan 36 French, straight, A/S, Värlöse, Denmark) to allow undisturbed blood flow from the hepatic veins via the caval vein to the heart–lung machine. After the 1-h perfusion period, the liver was flushed during approximately 10 min with 3 l Gelofusine. Thereafter, all catheters and clamps were removed and all incisions were closed. To prevent possible post-operative L-PAM induced cholecystitis, a cholecystectomy was performed.

Leakage of perfusate into the systemic circulation was monitored by adding $^{99m}$Tc-pertechnetate ($^{99m}$Tc) to the isolated circuit and subsequent measurement of the level of radioactivity in both the systemic and isolated circuit as previously described (Runia et al, 1987; Marinelli et al, 1996). If no leakage was detected, L-PAM (freshly prepared solution) was administrated as a bolus to the isolated circuit. In case of unacceptable leakage (e.g. above 10% at the highest L-PAM dose level) during the perfusion period, the procedure was immediately stopped and the liver was flushed as mentioned above at the IHP technique.

### Post-operative care

All patients were monitored in the intensive care unit for at least 1 day after IHP. Liver and kidney function tests (ALAT, ASAT, bilirubin, alkaline phosphatase, γ-GT, LDH, creatinine, ureum), number of platelets and white blood cell count were measured frequently. Toxicity was determined according to WHO recommendations. Patients received G-CSF (Filgrastim/Neupogen®, Amgen B.V., Breda, The Netherlands) in case of grade 3–4 leukopenia and routinely when treated with 3.0 mg $kg^{-1}$ L-PAM from 1 day after IHP until after the nadir.

### L-PAM pharmacokinetics

Heparinized samples for L-PAM measurements were taken at regular intervals from the perfusion medium and from the systemic circulation. Samples were centrifuged for 10 min at 750 ***g***, and the supernatant was stored at –80°C until analysis. In 12 cases, tumour and/or liver biopsies were taken within 30 min after IHP treatment, and immediately frozen in liquid nitrogen. All samples were analysed for L-PAM concentrations by an HPLC assay (Chang et al, 1978). The areas under the concentration-time curves (AUC) were calculated with the trapezoidal rule. The amount of L-PAM in tissue samples was expressed as μg $g^{-1}$ wet tissue.

### Treatment evaluation

Objective tumour responses were obtained by follow-up CT scans of the liver approximately 3 months after IHP treatment. Four patients were excluded because either no post-operative CT scans were available or tumour measurements were in view of the large amount of metastases unrealistic. A complete response (CR) was defined as a total disappearance of all lesions; a partial remission (PR) was defined as at least a 50% reduction in tumour size (the sum of the product of the perpendicular diameters of all measurable lesions); a stable disease (SD) was defined as less than 50% tumour size regression or less than 25% progression, and progressive disease (PD) was defined as more than 25% progression. Moreover, CEA levels were measured before and after IHP treatment.

### Statistics

All data were analysed using SPSS (version 9.0) software and presented as mean ± s.d. For all statistical analyses, a *P*-value < 0.05 was considered statistically significant. Correlation coefficients were calculated using the non-parametric Spearman's test.

### RESULTS

### Patient characteristics and IHP technique

Half of the patients treated in this study were staged Dukes' D at time of primary tumour resection. Mean age of treated patients was 53 years (range 36–64), the majority (15) being male. Only three patients previously had received chemotherapy.

Based on the L-PAM dose level, a maximum percentage of leakage (e.g. 10% $^{99m}$Tc leakage at dose level 3.0 mg $kg^{-1}$) was set beforehand, above which the perfusion should be terminated.

**Table 1** IHP parameters

| Parameter | With intra-caval shunt | | With veno-venous bypass | |
|---|---|---|---|---|
| | Mean ± s.d. | Range | Mean ± s.d. | Range |
| Flow rate (ml $min^{-1}$) | | | | |
| Hepatic artery | 403 ± 160 | 190–800 | 502 ± 86 | 400–621 |
| Portal vein | 492 ± 148 | 210–800 | 393 ± 66 | 315–460 |
| Total | 895 ± 146 | 595–1210 | 895 ± 145 | 715–1051 |
| Pressure, mmHg | | | | |
| Hepatic artery | 132 ± 36 | 80–200 | 164 ± 38 | 120–207 |
| Portal vein | 36 ± 12 | 10–70 | 37 ± 9 | 30–48 |
| Leakage (%) | 15 ± 14 | 0–41 | 7 ± 5 | 2–15 |
| Duration of perfusion, min | 51 ± 13 | 25–60 | 48 ± 16 | 30–60 |

© 2000 Cancer Research Campaign

**Table 2** Melphalan pharmacokinetics, perfusion and response parameters

| Dose level (mg kg$^{-1}$) | Dose total (mg) | Leakage (%) | Perfusion duration min | Peak L-PAM systemic (μg ml$^{-1}$) | Peak L-PAM perfusate (μg ml$^{-1}$) | AUC systemic (h μg ml$^{-1}$) | AUC perfusate (h μg ml$^{-1}$) | Tumour L-PAM (μg g$^{-1}$) | Liver L-PAM (μg g$^{-1}$) | CEA before IHP (μg ml$^{-1}$) | CEA after IHP (μg ml$^{-1}$) | Decrease in CEA (%[c]) | CT response |
|---|---|---|---|---|---|---|---|---|---|---|---|---|---|
| 0.5 | 35 | 4 | 60 | 0.4 | 5.1 | 0.3 | 3.1 | | | 1026 | 285.0 | 72 | SD |
| 0.5 | 35 | 0 | 60 | 0.1 | 6.0 | 0.1 | 3.8 | | | 5.2 | 3.4 | 35 | PD |
| 0.8 | 45 | 1 | 60 | 1.5 | 5.7 | 1.4 | 4.1 | | | 16.3 | 2.8 | Normal | PD |
| 1.0 | 70 | 2 | 60 | 0.4 | 14.0 | 0.4 | 6.8 | | | 970 | 7.2 | 99 | PR |
| 1.0 | 57 | 5 | 60 | 0.7 | 13.8 | 0.4 | 7.1 | | | 14 | 11.0 | 21 | PD |
| 1.0 | 68 | 18 | 60 | 0.8 | 9.2 | 0.6 | 6.0 | | | 35 | 3.1 | 91 | |
| 1.0[a] | 65 | 7 | 60 | 0.4 | 9.5 | 0.4 | 4.7 | 1.5 | 1.4 | 14 | 3.5 | 75 | |
| 1.3 | 78 | 2 | 60 | 0.9 | 12.5 | 0.6 | 7.4 | | | 34 | 8.1 | 76 | PD |
| 1.5[b] | 130 | 20 | 60 | 2.9 | 18.2 | 1.3 | 8.9 | | | | | | |
| 1.5 | 140 | 27 | 25 | 1.7 | 12.2 | 1.2 | 2.3 | 4.8 | 2.3 | 117 | 36.0 | 69 | SD |
| 1.7 | 154 | 20 | 30 | 4.2 | 54.1 | 1.5 | 9.5 | 1.7 | 11.6 | 4.9 | 1.9 | Normal | SD |
| 1.7 | 150 | 37 | 30 | 1.8 | 18.0 | 1.4 | 4.0 | | 4.4 | 30 | 5.1 | 83 | PD |
| 1.8 | 118 | 15 | 60 | 1.0 | 16.2 | 0.5 | 10.3 | | | 1.4 | 0.8 | | CR |
| 1.8 | 128 | ND | 60 | 1.1 | 12.4 | 0.5 | 6.6 | 0.0 | 2.1 | 27 | 2.8 | Normal | |
| 2.0 | 121 | 9 | 60 | 2.0 | 15.2 | 0.9 | 8.4 | | 3.3 | 9 | 2.2 | Normal | PR |
| 2.0 | 157 | 23 | 28 | 1.9 | 18.6 | 1.1 | 5.8 | | | 3.3 | 1.5 | Normal | SD |
| 2.0 | 180 | 1 | 60 | 0.9 | 21.0 | 0.4 | 11.5 | | 2.2 | 51 | 30.0 | 41 | SD |
| 2.5[b] | 250 | 41 | 55 | 4.5 | 38.4 | 3.1 | 18.9 | 5.5 | 6.0 | | | | |
| 3.0 | 187 | 6 | 60 | 0.7 | 29.7 | 0.4 | 17.1 | 13.5 | 14.3 | 229 | 16.0 | 93 | PR |
| 3.0 | 226 | 35 | 46 | 1.1 | 34.6 | 0.6 | 14.5 | | | 138 | 17.0 | 88 | PD |
| 3.0[a] | 190 | 8 | 30 | 1.3 | 32.0 | 1.6 | 10.4 | | | 38 | 1.1 | Normal | PR |
| 3.0[a] | 225 | 2 | 60 | 0.7 | 40.0 | 1.4 | 24.9 | | 15.7 | 11 | 1.6 | Normal | SD |
| 3.0[a] | 192 | 15 | 30 | 2.0 | 56.7 | 2.3 | 13.3 | 11.5 | 5.6 | 2574 | 170.0 | 93 | |
| 4.0[a,b] | 300 | 3 | 60 | 1.1 | 53.7 | 1.0 | 33.8 | 20.8 | 9.1 | | | | |

[a]Means that we used the IHP technique with veno-venous bypass. [b]Means patient died as a result of IHP treatment. L-PAM, melphalan; ND, not determined; CR, complete remission; PR, partial remission; SD, stable disease; PD, progressive disease. [c]Means normal CEA value ≤3.0 μg ml$^{-1}$

*© 2000 Cancer Research Campaign*

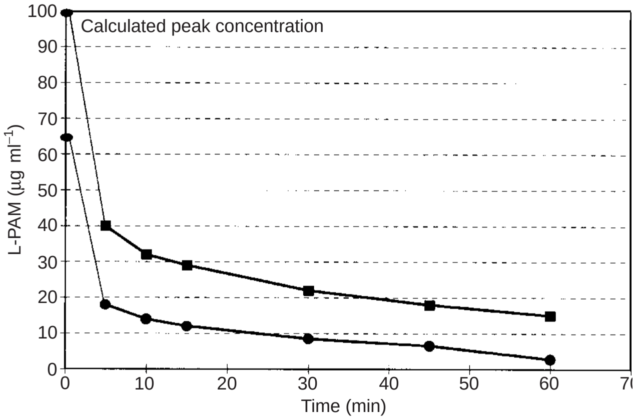

**Figure 2** Concentration of L-PAM in perfusate during the one-hour perfusion period after addition of either 1.5 mg kg$^{-1}$ (●) or 3.0 mg kg$^{-1}$ (■) L-PAM to the isolated circuit. The calculated L-PAM peak concentration is indicated

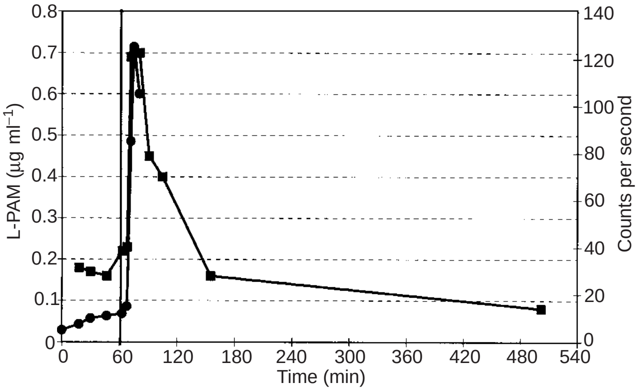

**Figure 3** Concentration of L-PAM (■) in the systemic circulation during and after the IHP procedure after addition of 3.0 mg kg$^{-1}$ L-PAM to the isolated circuit. Moreover, a typical pattern of the signal of $^{99m}$Tc labelled red blood cells (●) in the systemic circulation is shown. A peak $^{99m}$Tc level of 120 counts in the systemic circulation resembles a calculated leakage percentage of approximately 4–5%

**Table 3** Toxicity according to WHO criteria

| Parameter | Grade 0 | | Grade 1 | | Grade 2 | | Grade 3 | | Grade 4 | |
|---|---|---|---|---|---|---|---|---|---|---|
| | No. | % | No. | % | No. | % | No. | % | No. | % |
| Leucocyte | 15 | 63 | 2 | 8 | 2 | 8 | 1 | 4 | 3 | 13 |
| Platelets | 14 | 59 | 3 | 12 | 1 | 4 | 2 | 8 | 4 | 17 |
| Creatinine | 20 | 80 | 2 | 8 | 2 | 8 | 1 | 4 | 0 | 0 |
| ALAT | 14 | 61 | 6 | 26 | 2 | 9 | 0 | 0 | 1 | 4 |
| ASAT | 20 | 87 | 2 | 9 | 0 | 0 | 0 | 0 | 1 | 4 |
| Bilirubin | 10 | 44 | 5 | 22 | 4 | 17 | 3 | 13 | 1 | 4 |

Hepatotoxicity parameters (ALAT, ASAT, bilirubin) were scored from day 7 after perfusion. For hepatotoxicity, 23 patients were evaluated because one patient died within 7 days after IHP.

Duration of IHP was aimed at 60 min, but as a result of leakage from the isolated circuit to the systemic circulation, perfusion duration was prematurely stopped in eight cases. Based on this relatively high incidence of systemic leakage, we decided after 19 patients to change the IHP procedure, applying an external circuit to bypass the liver (Figure 1). In the next five patients, the perfusion procedure was stopped earlier in two cases. The present series is too small to derive definite conclusions whether leakage can be better controlled by applying the IHP technique with veno-venous bypass. However, it was recently reported that by using a similar IHP technique, only in two of 34 treated patients systemic leakage of perfusate was detected (Alexander et al, 1998). Perfusion parameters are listed in Table 1. Total mean perfusion flow did not differ significantly among the two different IHP techniques applied (being 895 ml min$^{-1}$ in both cases), and also the mean of all other perfusion parameters did not differ significantly. Therefore, we considered all patients as one group.

The surgical procedure (including the 1-h perfusion period) took approximately 5 h (range 4–7.5 h). Blood loss during the operation was considerable and ranged from 1.5 to 10 l. All patients stayed at least 1 day (standard protocol) in the intensive care unit, with a mean duration of 4 days (range 1–36 days). Mean hospital stay was 17 days (range 8–53 days). Of all patients treated, 50% were discharged from the hospital within 14 days after the perfusion. Three patients died as a result of the IHP treatment (14% mortality rate).

### L-PAM pharmacokinetics

Data on individual L-PAM pharmacokinetics are listed in Table 2. From all patients treated by IHP, L-PAM concentrations were measured in both the isolated circuit and the systemic circulation and the AUC was calculated. The AUC in the perfusate reflects the L-PAM exposure to the liver and metastases. A statistically significant correlation was found between the L-PAM dose and the peak concentration ($R = 0.86$, $P < 0.001$) and the AUC ($R = 0.82$, $P < 0.001$) of L-PAM in perfusate respectively. Figure 2 shows two examples of the L-PAM concentration in the perfusate at two dose levels (respectively 1.5 mg kg$^{-1}$ and 3.0 mg kg$^{-1}$): a twofold increase in L-PAM dose translated into a similar increase in peak L-PAM concentration in perfusate as measured 5 min after addition of L-PAM. Based on the estimated peak L-PAM concentration in perfusate, a biphasic decline in L-PAM concentrations was found in perfusate, indicating a rapid uptake phase followed by a much slower elimination phase.

The mean peak concentration and AUC of L-PAM in perfusate in patients treated at the highest tolerable dose level (3.0 mg $kg^{-1}$) were 38.6 ± 10.8 µg $ml^{-1}$ and 16.6 ± 5.5 h µg $ml^{-1}$ respectively, whereas the mean systemic peak plasma concentration and AUC were 1.1 ± 0.5 µg $ml^{-1}$ and 1.3 ± 0.8 h µg $ml^{-1}$. These data demonstrate that at the target side, the peak L-PAM concentration and the AUC L-PAM were respectively 35- and 13-fold higher than in the systemic circulation, and clearly indicate that a complete vascular isolation of the liver can be obtained for a prolonged period of time.

The peak L-PAM concentration in the systemic circulation was always observed after termination of the IHP procedure. In the majority of cases almost no systemic leakage of L-PAM was observed during the IHP treatment; a typical case is shown in Figure 3. This pattern corresponds with the level of radioactivity in the systemic circulation: after the washout period when the clamps were removed from the caval vein, approximately 4–5% of the total dose $^{99m}$Tc, was re-distributed into the systemic circulation (Figure 3). All $^{99m}$Tc is supposed to be bound to red blood cells (Runia et al, 1987). Because of the identical re-distribution pattern of L-PAM and the $^{99m}$Tc-labelled red blood cells, this residual L-PAM probably originates from vessels not sufficiently flushed after the perfusion period.

It was possible to collect tumour and liver tissue biopsies from a limited number of patients ($n$ = 8) treated at various L-PAM dosages. The amount of L-PAM detected by HPLC in tumour and liver tissue ranged from 0 to 20.8 and 1.4–15.7 µg $g^{-1}$ respectively. A statistically significant correlation was found between the amount of L-PAM in tumour and liver tissue and the dose level (tumour: R = 0.83; $P$ = 0.011, liver: R = 0.65; $P$ = 0.022) or AUC perfusate (tumour: R = 0.76; $P$ = 0.03, liver: R = 0.71; $P$ = 0.01) of L-PAM.

### Toxicity of L-PAM

With respect to bone marrow-related toxicity, four patients developed grade 3–4 leukopenia and six patients developed grade 3–4 thrombocytopenia (Table 3). Most patients had only a small drop in leukocytes and the nadir (mean 4.8 ± 2.8 $10^9$ $l^{-1}$, range 0.1–9.3) was usually observed approximately 8–9 days after IHP. A significant correlation was found between the nadir in leukocytes and the dose level of L-PAM (R = –0.47, $P$ = 0.024). Two of the three patients who developed a grade 4 leukopenia, had the two highest systemic L-PAM AUC values, making it likely that L-PAM was responsible for the observed bone marrow toxicity. One of these two patients died 11 days after IHP as a result of sepsis, which was probably indirectly caused by massive systemic leakage (41%) occurring shortly before termination of isolation. In this patient, who was treated with 250 mg total dose L-PAM, a concentration of 4.5 µg $ml^{-1}$ L-PAM was measured in the systemic circulation (being the highest concentration measured in our patient population). The patient who developed a grade 3 leukopenia died 5 days after the IHP treatment as a result of toxic shock. The observed bone marrow toxicity might be related to systemic L-PAM leakage because in this patient also a high systemic L-PAM concentration of 2.9 µg $ml^{-1}$ was measured.

The only patient who was treated with 4.0 mg $kg^{-1}$ L-PAM developed a grade 4 leukopenia shortly after IHP. No high systemic L-PAM concentrations were measured (Table 2), which was in accordance with $^{99m}$Tc measurements during perfusion,

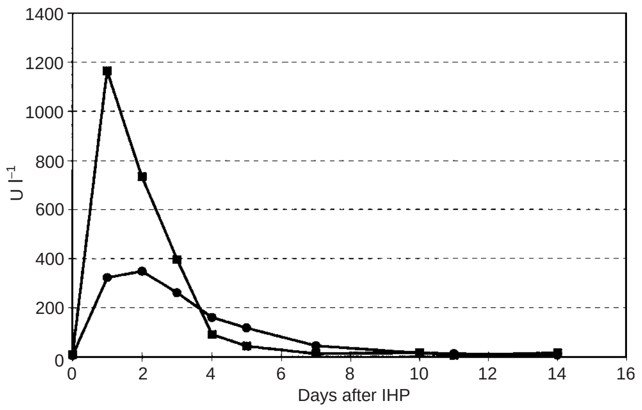

**Figure 4** The amount of serum ALAT (●) and ASAT (■) after the IHP procedure with 3.0 mg $kg^{-1}$ L-PAM. A typical case is shown indicating no systemic leakage. This patient died 8 days after the IHP procedure, showing acute liver and kidney failure, adult respiratory distress syndrome and coagulation disorders. This patient had approximately 60% of the liver involved with tumour tissue, which most likely was an additional risk factor. At autopsy, all tumour tissue with surrounding liver tissue was found to be necrotic. Therefore, the observed bone marrow toxicity and development of multiple-organ failure in this patient could be due to the release of cytokines like TNF from the liver.

During the first 2 days after IHP, all patients had a minor to marked increase in the serum level of bilirubin (mean 71 ± 76 µmol $l^{-1}$, range 13–372), LDH (mean 3034 ± 4397 U $l^{-1}$, range 190–17 600), ASAT (mean 466 ± 833 U $l^{-1}$, range 23–3900) and ALAT (mean 319 ± 337 U $l^{-1}$, range 22–1078), which returned to normal within approximately 1 week after IHP (Figure 4). No correlation was found between L-PAM perfusion parameters and the peak level of bilirubin, LDH, ASAT and ALAT, indicating that the observed hepatotoxicity during the first days post-IHP was caused in part by the perfusion procedure itself, including the physical and vascular manipulations of the liver. Therefore, as recently suggested (Alexander et al, 1998), elevations in liver enzymes and bilirubin beyond 7 days after the IHP procedure were considered as L-PAM-related. Based on both bilirubin and liver enzyme levels, grade 4 hepatotoxicity was observed only in the patient who was treated with 4.0 mg $kg^{-1}$ L-PAM. In the majority of patients, grade 0 and 1 hepatotoxicity was observed (Table 3).

### Tumour response and patient survival

In this series, 17 patients were evaluable for measurement of tumour response and the overall response rate was 29% (Table 2: one complete remission, four partial remissions, six stable diseases and six progressive cases). All patients had a substantial decrease in carcinoembryonic antigen (CEA) after IHP ranging from 21% to normalization of the CEA level (CEA ≤ 3.0 µg $ml^{-1}$), which was observed in seven patients (Table 2). Patient survival after IHP treatment ranged from 3.7 up to 85 months, but no correlation was found between CEA decrease and survival duration. Median and mean patient survival of the whole series ($n$ = 21) after IHP were 19 and 27 months respectively, and 23 and 31 months respectively after the diagnosis of liver metastases. A statistically significant

 © 2000 Cancer Research Campaign correlation was found between patient survival after IHP and dose level of L-PAM (R = 0.52; $P = 0.02$) and perfusate AUC (R = 0.47; $P = 0.03$) respectively.

### DISCUSSION

In the present study we evaluated L-PAM dose-escalation by IHP as treatment for patients with colorectal cancer metastases confined to the liver. Our data demonstrate that increasing the L-PAM dose resulted in a statistically significant increase in AUC and peak L-PAM concentrations in perfusate as well as increased L-PAM uptake by tumour tissue. Compared to isolated limb perfusion for melanoma, peak L-PAM concentrations, perfusate AUC and tumour concentrations were in the same range (Klaase et al, 1994). However, the mean perfusate peak L-PAM concentration and AUC at the highest tolerable dose level (3.0 mg $kg^{-1}$) were approximately 30- and fivefold higher respectively, than reported for high dose (80–157 mg $m^{-2}$) oral L-PAM and bone marrow transplantation (Choi et al, 1989; Boros et al, 1990). Therefore, IHP enabled exposure of the liver and metastases to much higher L-PAM concentrations than achievable after systemic administration.

IHP is a technically difficult way of drug delivery with morbidity and mortality associated with the operative procedure. A weak but statistically significant correlation was found between the L-PAM dose level and the nadir in white blood cells after IHP. It is noteworthy that no correlation was found between L-PAM concentration parameters and liver enzyme disturbances, indicating that the observed hepatotoxicity during the first days after the IHP procedure was caused by the IHP procedure itself rather than by high drug levels – with possible exception of the only patient treated with 4.0 mg $kg^{-1}$ who died at day 8 after IHP as a result of multiple-organ failure. In retrospect, the sudden death of this patient might be explained by the fact that his liver contained a high tumour load. A similar finding was described by Hafstrom and colleagues (Hafstrom et al, 1994), who treated patients by IHP with L-PAM and cisplatin under hyperthermic conditions: from the patients who had more than 50% of the liver occupied by cancer, 25% died within 30 days after IHP due to multiple-organ failure. We concluded from these toxicity data that a maximum dose of L-PAM for delivery via IHP is approximately 3.0 mg $kg^{-1}$.

In most clinical IHP studies, livers were perfused arbitrarily for 1 h. Pharmacokinetic analysis of L-PAM in perfusate showed a biphasic decline and we demonstrated that after the initial uptake phase (approximately 10–15 min), the inflow and outflow L-PAM concentrations were similar (Vahrmeijer et al, 1996), indicating that the liver removed little L-PAM after the initial uptake phase. These pharmacokinetic data suggest that in case of L-PAM, perfusion duration can be shortened to approximately 15–30 min.

At present, it is unclear to what extent the perfusate temperature, perfusion flow rate and pressure in the hepatic artery influence tumour L-PAM uptake. In our study the mean flow rate and pressure in the hepatic artery inflow catheter were 502 ml $min^{-1}$ and 164 mmHg respectively; in the aforementioned study by Alexander and colleagues (Alexander et al, 1998), who used a combination of 1.5 mg $kg^{-1}$ L-PAM and 1.0 mg TNF, the pressure was in the same range (159 mmHg) but the flow rate was substantially higher (844 ml $min^{-1}$). Notably, the reported overall response rate in the latter study was higher than in our study (76% vs 29%). The addition of TNF, or the higher perfusion flow rate in the hepatic artery, or the fact that they used mild hyperthermia (central hepatic temperature of 39.9°C), or combinations thereof might contribute to this remarkable difference in response rate.

The present series is too small to derive definite conclusions with respect to response parameters, but data suggest that increasing the L-PAM dose translates into prolonged patient survival. Overall median survival after IHP in our series was 19 months ($n = 21$) and this is comparable to data that we obtained with mitomycin C (Marinelli et al, 1996). Although no adjuvant systemic treatment was given, the majority of patients received standard chemotherapy following progression. With respect to patient survival, until now best results with other techniques have been obtained with fluoropyrimidines delivered as a continuous infusion into the hepatic artery. Reported median survival durations from phase II trials of hepatic artery infusion (HAI) of fluoropyrimidines (in patients who complied with the same selection criteria as used for IHP studies) range from 12 to 26 months (Kemeny et al, 1994, 1995; Vahrmeijer et al, 1995; Meta Analysis Group in Cancer, 1996). As soon as survival data from ongoing phase II IHP studies become available, comparisons can be made with the large amount of data obtained in HAI studies.

In the present series, five patients were treated with 3.0 mg $kg^{-1}$ L-PAM and mean survival of this subgroup was 42 months (range 13–70 months). Moreover, two out of four evaluable patients had a partial tumour remission (Table 2). Based on these initial favourable results, we started a phase II study of IHP with 200 mg total dose L-PAM. A fixed total dose of 200 mg L-PAM was chosen because this dose was well tolerated in the present study. Moreover, in the ongoing phase II study all patients receive granulocyte colony-stimulating factor (Filgrastim/Neupogen®) to protect against leukopenia. Irrespective of the outcome of latter study, it is clear that IHP would gain much wider interest if the whole procedure could be simplified. In that context, several investigators have proposed less invasive techniques based upon balloon catheterization (Curley et al, 1994; Ravikumar et al, 1994; van Ijken et al, 1998).

In conclusion, IHP is technically feasible. The largest proportion of L-PAM was taken up within the first 10–15 min after addition of L-PAM to the perfusate and within the dose range evaluated, higher doses of L-PAM indeed lead to higher concentrations of L-PAM in the liver metastases. Doses up to 3.0 mg $kg^{-1}$ did not lead to VOD or other serious hepatotoxicities. The current ongoing phase II study will provide detailed information on response of liver metastases to this treatment modality and the impact on patient survival. However, it should be re-emphasized that IHP is still an experimental treatment modality with at present no place in routine clinical practice. Therefore, progress should be made to develop a simplified procedure, e.g. a totally percutaneously applicable IHP technique.

### ACKNOWLEDGEMENTS

This study was supported by grant RUL 93-495 from the Dutch Cancer Society. We thank R van Gijn for L-PAM analysis in biological samples, Dr J Hermans for statistical analysis, and the contribution of F Tijl, head of the extra-corporeal circulation unit.

### REFERENCES

Aigner KR, Walther H and Link KH (1988) Isolated liver perfusion with MMC/5-FU: surgical technique, pharmacokinetics, clinical results. *Contr Oncol* **29**: 229–246

Alexander HR, Bartlett DL, Libutti SK, Fraker DL, Moser T and Rosenberg SA

© 2000 Cancer Research Campaign

(1998) Isolated hepatic perfusion with tumour necrosis factor and melphalan for unresectable cancers confined to the liver. *J Clin Oncol* **16**: 1479–1489

Borel Rinkes IHM, de Vries MR, Jonker AM, Swaak TJ, Hack CE, Nooyen PT, Wiggers T and Eggermont AMM (1997) Isolated hepatic perfusion in the pig with TNF-alpha with and without melphalan. *Br J Cancer* **75**: 1447–1453

Boros L, Peng YM, Alberts DS, Asbury RF, Goodman TL, Penn TE and Hickox DE (1990) Pharmacokinetics of very high-dose oral melphalan in cancer patients. *Am J Clin Oncol* **13**: 19–22

Chang SY, Alberts DS, Farquhar D, Melnick LR, Walson PD and Salmon SE (1978) Hydrolysis and protein binding of melphalan. *J Pharm Sci* **67**: 682–684

Choi KE, Ratain MJ, Williams SF, Golick JA, Beschorner JC, Fullem LJ and Bitran JD (1989) Plasma pharmacokinetics of high-dose oral melphalan in patients treated with trialkylator chemotherapy and autologous bone marrow reinfusion. *Cancer Res* **49**: 1318–1321

Curley SA, Newman RA, Dougherty TB, Fuhrman GM, Stone DL, Mikolajek JA, Guercio S, Guercio A, Carrasco CH and Kuo MT (1994) Complete hepatic venous isolation and extracorporeal chemofiltration as treatment for human hepatocellular carcinoma: a phase I study. *Ann Surg Oncol* **1**: 389–399

Garcia ST, McQuillan A and Panasci L (1988) Correlation between the cytotoxicity of melphalan and DNA crosslinks as detected by the ethidium bromide fluorescence assay in the F1 variant of B16 melanoma cells. *Biochem Pharmacol* **37**: 3189–3192

Hafstrom, LR, Holmberg SB, Naredi PL, Lindner PG, Bengtsson A, Tidebrant G and Schersten TS (1994) Isolated hyperthermic liver perfusion with chemotherapy for liver malignancy. *Surg Oncol* **3**: 103–108

Kemeny N (1995) Regional chemotherapy of colorectal cancer. *Eur J Cancer* **31A**: 1271–1276

Kemeny N, Conti JA, Cohen A, Campana P, Huang Y, Shi WJ, Botet J, Pulliam S and Bertino JR (1994) Phase II study of hepatic arterial floxuridine, leucovorin, and dexamethasone for unresectable liver metastases from colorectal carcinoma. *J Clin Oncol* **12**: 2288–2295

Klaase JM, Kroon BB, Beijnen JH, van Slooten GW and van Dongen JA (1994) Melphalan tissue concentrations in patients treated with regional isolated perfusion for melanoma of the lower limb. *Br J Cancer* **70**: 151–153

Leff RS, Thompson JM, Johnson DB, Mosley KR, Daly MB, Knight WA, Ruxer RL and Messerschmidt GL (1986) Phase II trial of high-dose melphalan and autologous bone marrow transplantation for metastatic colon carcinoma. *J Clin Oncol* **4**: 1586–1591

Marinelli A, Dijkstra FR, van Dierendonck JH, Kuppen PJK, Cornelisse CJ and van de Velde CJH (1991*a*) Effectiveness of isolated liver perfusion with mitomycin C in the treatment of liver tumours of rat colorectal cancer. *Br J Cancer* **64**: 74–78

Marinelli A, van Dierendonck JH, van Brakel GM, Irth H, Kuppen PJK, Tjaden UR and van de Velde CJH (1991*b*) Increasing the effective concentration of melphalan in experimental rat liver tumours: comparison of isolated liver perfusion and hepatic artery infusion. *Br J Cancer* **64**: 1069–1075

Marinelli A, de Brauw LM, Beerman H, Keizer HJ, van Bockel JH, Tjaden UR and van de Velde CJH (1996) Isolated liver perfusion with mitomycin C in the treatment of colorectal cancer metastases confined to the liver. *Jpn J Clin Oncol* **26**: 341–350

Meta Analysis Group in Cancer (1996) Reappraisal of hepatic arterial infusion in the treatment of nonresectable liver metastases from colorectal cancer. *J Natl Cancer Inst* **88**: 252–258

Oldhafer KJ, Lang H, Frerker M, Moreno L, Chavan A, Flemming P, Nadalin S, Schmoll E and Pichlmayr R (1998) First experience and technical aspects of isolated liver perfusion for extensive liver metastasis. *Surgery* **126**: 622–631

Ravikumar TS, Pizzorno G, Bodden W, Marsh J, Strair R, Pollack J, Hendler R, Hanna J and D'Andrea E (1994) Percutaneous hepatic vein isolation and high-dose hepatic arterial infusion chemotherapy for unresectable liver tumours. *J Clin Oncol* **12**: 2723–2736

Runia RD, de Brauw LM, Kothuis BJ, Pauwels EK and van de Velde CJH (1987) Continuous measurement of leakage during isolated liver perfusion with a radiotracer. *Int J Rad Appl Instrum B* **14**: 113–118

Vahrmeijer AL, van Dierendonck JH and van de Velde CJH (1995) Treatment of colorectal cancer metastases confined to the liver. *Eur J Cancer* **31A**: 1238–1242

Vahrmeijer AL, Snel CA, Steenvoorden DP, Beijnen JH, Pang KS, Schutrups J, Tirona R, Keizer HJ, van Dierendonck JH, van de Velde CJH and Mulder GJ (1996) Lack of glutathione conjugation of melphalan in the isolated in situ liver perfusion in humans. *Cancer Res* **56**: 4709–4714

van Ijken MGA, de Bruijn EA, de Boeck G, ten Hagen TLM, van der Sijp JRM and Eggermont AMM (1998) Isolated hypoxic hepatic perfusion with tumour necrosis factor-alpha, melphalan, and mitomycin C using balloon catheter techniques: a pharmacokinetic study in pigs. *Ann Surg* **228**: 763–770

*© 2000 Cancer Research Campaign*
